# Protective antibodies against enterotoxigenic *Escherichia coli* are generated from heat-labile toxoid vaccination and exhibit subject- and vaccine-specific diversity

**DOI:** 10.1007/s00430-025-00817-3

**Published:** 2025-02-11

**Authors:** Milton Maciel, Jordan C. Scott, Robin L. Baudier, John D. Clements, Renee M. Laird, Ramiro L. Gutiérrez, Chad K. Porter, Elizabeth B. Norton

**Affiliations:** 1 Operationally Relevant Infections Department, Naval Medical Research Command, Silver Spring, MD USA; 2 Henry M. Jackson Foundation for the Advancement of Military Medicine, Inc, Bethesda, MD USA; 3 Department of Microbiology and Immunology, Tulane University School of Medicine, New Orleans, LA USA; 4 Translational and Clinical Research Department, Naval Medical Research Command, Silver Spring, MD USA; 5 Present Address: Division of AIDS, National Institute of Allergy and Infectious Diseases, National Institutes of Health, Rockville, MD USA; 6 Present Address: Biostatistics and Design Program, Oregon Health and Sciences University, Portland, USA; 7 Present Address: Diarrheal Disease Research Branch, Walter Reed Army Institute of Research, Silver Spring, USA; 8 Present Address: State University of New York Upstate Medical University, Syracuse, USA

**Keywords:** ETEC, Heat-labile toxin, Antibodies, Vaccination, Diarrheal disease, Controlled human challenge

## Abstract

Heat-labile toxin (LT) from enterotoxigenic *Escherichia coli* (ETEC) is an important pathogenic protein. Anti-LT antibodies (Abs) induced by vaccination can neutralize the toxin and potentially prevent diarrheal secretion from ~ 60% of ETEC strains expressing LT. However, only superficial investigation of the anti-toxin response is usually conducted in clinical trials. Here, we utilized human serum samples from two clinical trials performed to assess safety, immunogenicity and protection in a controlled human infection model with a LT + ST + CFA/I + H10407 ETEC strain. These Phase 1 and Phase 2b clinical trials explored a prototype ETEC adhesin (CfaE) and a chimeric adhesin-toxoid protein (dscCfaE-CTA2/LTB5) delivered intradermally or transcutaneously with a mutated form of LT (mLT) as an adjuvant. Serum samples were tested for antigen-specific IgG or IgA Abs by immunoblot, enzyme-linked immunosorbent assay (ELISA), or functional neutralizing Abs using LT holotoxin, LTA or LTB subunits. Abs to both LT subunits were present, but the response to each was altered by vaccine formulation, dose, and delivery routes as well as subject. The anti-LT IgG response correlated best to neutralizing antibodies and protection from H10407 controlled challenge when compared to other measures including serum IgA or anti-fimbriae (CfaE) Abs. In addition, our results helped to explain cohort attack rate differences in naïve unvaccinated participants and we found higher anti-LTA IgG post-challenge significantly related to ETEC severity score. Thus, strategies generating and measuring immunity to the complete AB_5_ structure of LT and subunits are better determinant of assessing protective immunity against LT + or LT + ST + ETEC diarrheal secretion in humans.

**Supplementary Information:**

The online version contains supplementary material available at 10.1007/s00430-025-00817-3.

## Introduction

Enterotoxigenic *Escherichia coli* (ETEC) is an important human pathogen, causing significant diarrheal disease world-wide particularly in children under 5 years of age, travelers, or deployed military [1–6]. Intestinal fluid secretion is caused by either of two bacterial toxins, a protein called heat-labile toxin (LT) or a peptide called heat-stabile toxin (ST). Though regional strain differences in toxin expression are evident in cross country comparisons, it can be equally divided into thirds as those express LT, ST or both LT and ST [4, 7–10]. Since LT is both a protein and immunogenic, it can be recognized by antibodies (Abs) following natural infection or vaccination to neutralize its toxic effects [4, 11–14].

The LT toxin is an 85 kDa protein with an AB_5_ structure. The 11.5 kDa B-subunit (LTB) forms a pentamer and mediates ganglioside binding for receptor mediated endocytosis. The 28 kDa A-subunit (i.e., LTA) mediates intracellular enzymatic activity including cAMP release through its activation of adenylate cyclase. A few reports show that Abs can be generated to LTA after human infection or animal vaccination [15, 16]. In addition, Abs to either subunit can protect against toxin activities in vitro by inhibition of LT-induced cAMP accumulation in tissue culture or in vivo by blocking LT-induced intestinal secretion in mice [16]. However, most clinical testing has focused on detection of LTB Abs exclusively [12, 17–24].

In addition to secretory and immunogenic properties, LT is an adjuvant capable of boosting immunity to co-delivered antigens [24–27]. Genetic mutations have been introduced to divorce the toxicity of the molecule from its immunogenicity and adjuvanticity, including molecules like LT-R192G (mLT), and LT-R192G/L211A (dmLT) [28, 29]. LT, LTA or LTB subunits have also been explored for vaccine development individually or in fusion forms with other subunit antigens, particularly through LTB-antigen chimeras [30, 31]. Although a LT skin patch vaccine failed in Phase 3 clinical trials to protect vaccinated subjects from ETEC or all causes of moderate to severe diarrheal disease [32, 33]. More recently, clinical success has been achieved in Phase 1 and 2 studies with injected or oral ETEC vaccines containing mutated LT proteins [34–37] that mainly seek to boost immunity targeting both toxin and colonization factors of the ETEC bacterium. Different variants of LT antigens exist both naturally and as vaccine antigens. For example, animal ETEC isolates express a form of the toxin called type II LT (LT-IIa-c), that have only 16–20% similarity in amino acid sequences of their B-subunits while maintaining 52–56% similarity to A-subunits [38–40]. Different ETEC vaccine strategies use varying approaches of AB_5_ toxoids versus LTB-fusion antigens to induce anti-LT immunity. It is likely that the antigen form of toxin or toxoid exposure may also affect generation of Abs against LTA and LTB post-vaccination or infection and this may differentially impact the protection against diarrhea mediated by the anti-toxin response.

We have previously identified immunoprotective contributions of LTA and have purified holotoxin or subunit antigens that are available for clinical immunoassays to dissect the anti-toxin response. Here, we utilized these assays on samples from clinical trials that investigated different formulations based on a prototype ETEC adhesin vaccine targeting the CFA/I colonization factor using the subunit antigens dscCfaE or dscCfaE-CTA2/LTB5 (an adhesin-toxoid chimera) administered with or without the mLT by transcutaneous (TCI) or intradermal (ID) immunization. We hypothesized that a more detailed examination of the antibody responses to LT toxin may (i) provide a clearer rationale for selection of ETEC vaccine candidates for clinical development and (ii) confirm its role in protection from the 60% of ETEC strains expressing LT + or LT + ST + toxins.

## Materials and methods

*Serum Samples for Quantitative Research:* Deidentified human serum samples were obtained from completed Phase 1 (clinicaltrials.gov ID: NCT01644565) and Phase 2b (NCT01922856) previously published clinical trials[41, 42]. The two studies were performed sequentially to (1) assess safety, immunogenicity, and route and dose selection, and (2) test protection from oral challenge with a CFA/I + ETEC strain H10407 (for clinical design see Tables S1 and S2). Serum samples from the Phase 1 clinical trial were collected by venipuncture prior to immunization and on days 21, 42, 56 and 70, while samples from the Phase 2b trial were collected from vaccine group prior to immunization and on days 21, 42, 69, and 98. The unvaccinated control group was collected prior to challenge and four weeks later. Only samples from Phase 2b cohorts B and C, where the disease induced naïve subjects was consistent with prior studies [43], were used here. Commercially purchased human serum albumin from Sigma (H4522 lot# SLBJ1038V) was used as a negative control and to set the limit of detection in each assay. For testing purposes, samples were randomized and thawed in batches for immediate testing.

*Phase 2b challenge outcomes*: Values for moderate-to-severe diarrhea (MSD), ETEC severity score, and number of H10407 CFUs on day 2 and day 4 post challenge were previously reported [43] and are used here for outcome correlations.

*Antigens:* LT and LTA were purified using *E. coli* expression systems as previously described [16] Each lot of materials was verified by SDS-PAGE, patent mouse assay, or epithelial cell intoxication (i.e., cAMP, ELISA) prior to use in antibody detection assays.

*Immunoblot (IB).* LT IB were performed as previously reported [16] determining antibody binding to linear epitopes of LTA or LTB. Briefly, 1 μg LT was loaded into a 26-well 10% Bis–Tris (Bio-Rad) for SDS-PAGE and then transferred using Iblot® transfer stack, nitrocellulose, regular size (Invitrogen). Individual lanes were cut with a scalpel after temporary identification of proteins with Ponceau stain (0.1% Ponceau, 5% Acetic Acid). Each lane was blocked with in 5% BSA blocking buffer, and then stained with 1 μl human sample in 1 ml blocking buffer. After washing in PBS-Tween-20, the original blot was reconstructed on clear contact paper (Con-Tact Brand) before staining for with goat anti-human IgG-HRP (Invitrogen 627,120) or IgA-biotin (Southern Biotech 2050–08) and then Avidin-HRP (ebiosciences) were added. Blots were washed and then developed with ECL Western Blotting Substrate (Thermo Scientific) and an ImageQuant LAS 4000 biomolecular imager. Each contact paper also included a set of standard dilutions of purified human IgG (Pierce 31,154) or human IgA (Southern Biotech, 0155 K-01) that had been separately directly loaded onto nitrocellulose paper (Biorad) using Hybri-slot vacuum apparatus (Life Technologies). Final results were calculated using ImageJ with background subtracting as anti-LT or LTA Ig as A/B-band Ig density unit per ml serum sample. An external control serum as a negative control was used to set the limit of detection for assay.

*ELISA.* Serum samples were quantified for the presence of IgG and IgA Abs to recombinant purified A-subunit of LT toxin or recombinant purified LT holotoxin (containing both A- and B-subunits) by standard ELISA assay similar to previous studies[16]. Briefly, Linbro/Titertek 96-well U-shaped plates (MP Biomedicals 76–341-05) were coated with 1 μg/ml LT or 1.5 μg/ml LTA in carbonate coating buffer, pH 9.6. Plates were blocked with 1% BSA (KPL) and then 100 μl serial dilutions of each serum samples (starting at 1:100 dilution) were added for incubation. Washing occurred in between incubation steps with PBS-Tween-20. Plates were developed with anti-human IgG (Sigma A9544) or IgA-biotin (Southern Biotech 2050–08)/anti-biotin IgG-AKP (Sigma A7064) and pNPP-diethanolamine substrates. Absorbance at 405 nm was measured and then wells coated with dilutions of recombinant human IgG (Pierce 31,154) or IgA (Southern Biotech, 0155 K-01) were used as standards to calculate final results (e.g. IgG or IgA ELISA unit (EU)/ml). Anti-LTB or anti-CfaE ELISA results were included from previous testing[41].

*LT neutralization Assay.* Serum samples were quantified for presence of toxin neutralizing Abs by in vitro testing. Dilutions of serum samples were incubated for 24 h with 0.1–0.0001 μg of LT holotoxin prior to treatment of confluent Caco-2 cells, a human colonic epithelial cell line. After 18 h culture, cells were lysed and intracellular cAMP levels determined by cAMP ELISA detection kit (Parameter™ cAMP Assay, R&D Systems).

*Statistical analyses.* Data were compiled and analyzed using GraphPad Prism v10 and JMP Pro v.17. Data were expressed as means and standard error of the mean. Phase 1 antibody responses were analyzed with a two-way ANOVA with an uncorrected Fisher’s LSD post-test for each group to control cohort A-1 (1 μg CfaE ID). Correlations between neutralization and antibody responses were analyzed using Spearman’s rank correlation coefficient and expressed in a heat map with p-values reported testing the null hypothesis that the true correlation between the variables is zero*.* Phase 2 ELISA results were analyzed with paired one-way ANOVA with Dunnett post-test and paired T-test to indicated pre-vaccination/challenge timepoint. Correlations between ELISA results and MSD or severity score were tested with the Spearman rank correlation test to obtain Fisher’s Z transformed coefficients. Schematics of study design were created with Biorender.com. The ribbon structure of LT was created using PDB ID: 1LTS from a previous publication by Sixma, et al. [44].

## Results

We verified cross-reactivity levels for detection of human IgG and IgA in serum antigen-specific reactions and optimized the concentration of reagents for the assays. The anti-IgG detection Ab used for ELISA (Sigma) showed an average cross-reactivity of 8.7% against recombinant human IgA. The anti-IgG detection Ab used for immunoblot (Invitrogen) had an average cross-reactivity of 91.9% against human IgA. The IgA detection Ab (SouthernBiotech) employed for ELISA and immunoblot showed an average cross-reactivity < 0.3% against human IgG (e.g. Figure S1). This is distinct from the previously published IgA ELISA testing [41, 45] (e.g., for anti-LTB IgA testing), we used a human IgA antibody (from KPL) that had an average cross reactivity of > 50% to human IgG.

**Fig. 1:**
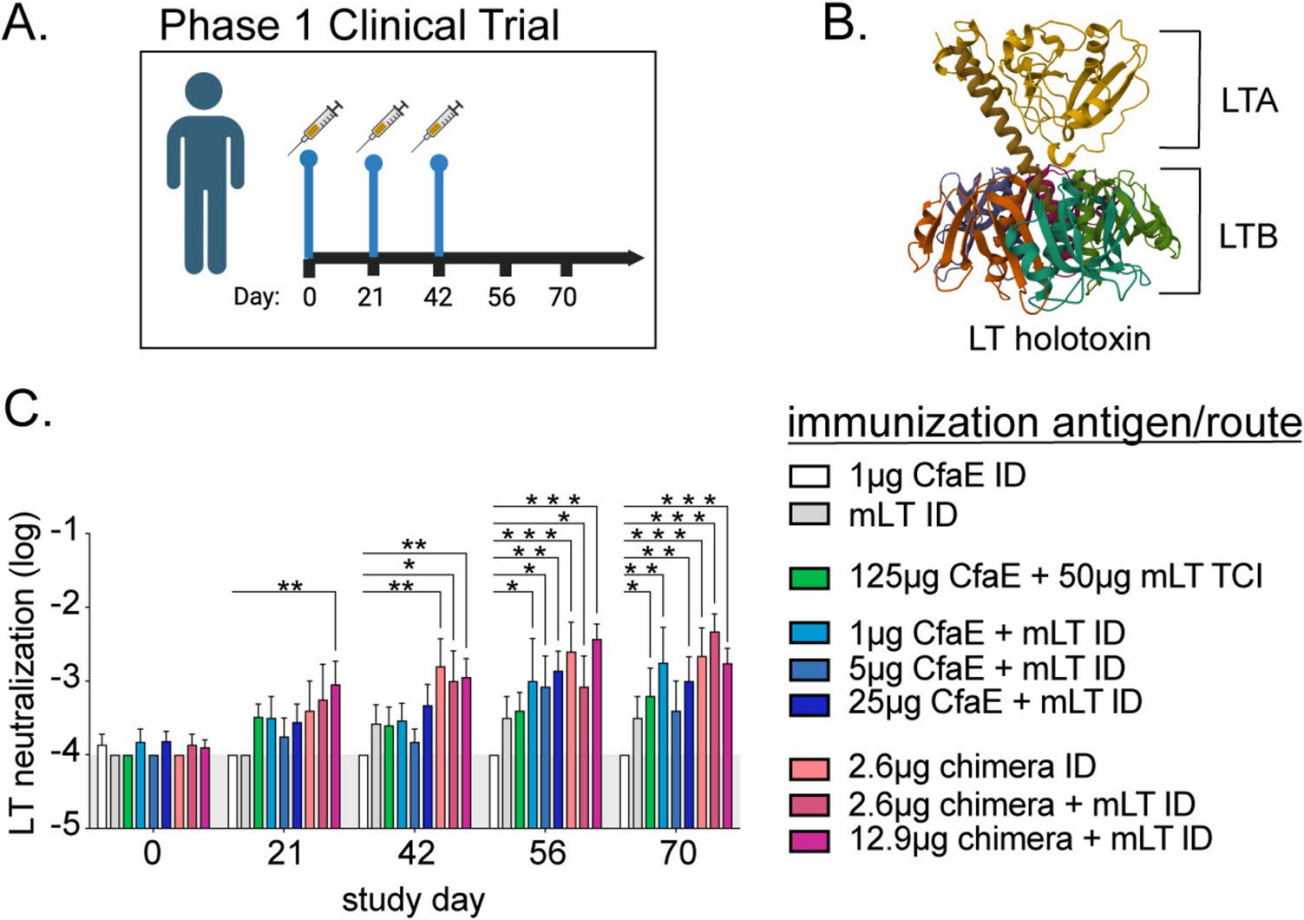
ETEC Phase 1 clinical trial resulted in increased serum in vitro LT toxin neutralizing antibody responses over time based on immunization group. **A** Schematic of the Phase 1 clinical trial with immunization on days 0, 21, and 42. Groups (n = 4–9) were immunized on days 0, 21, and 42 with various antigen combinations. mLT was dosed at 50 or 0.1 μg for, respectively, TCI or ID immunizations and chimera antigen contained an LTB antigen. Serum was collected on days 0, 21, 42, 56, and 70. **B** LT holotoxin ribbon diagram with subunits indicated. **C** Bar graph of serum toxin neutralization (log of μg LT neutralized using epithelial cell cAMP intoxication assay) by study day with immunization groups indicated. Bars at mean + SEM with significance indicated using two-way ANOVA with uncorrected Fisher’s LSD post-test to the 1 μg CfaE ID group (lacking any LT toxoid antigen) indicated as *P < 0.05, **P < 0.01, or ***P < 0.001. Gray area indicates the limit of detection

**Fig. 2:**
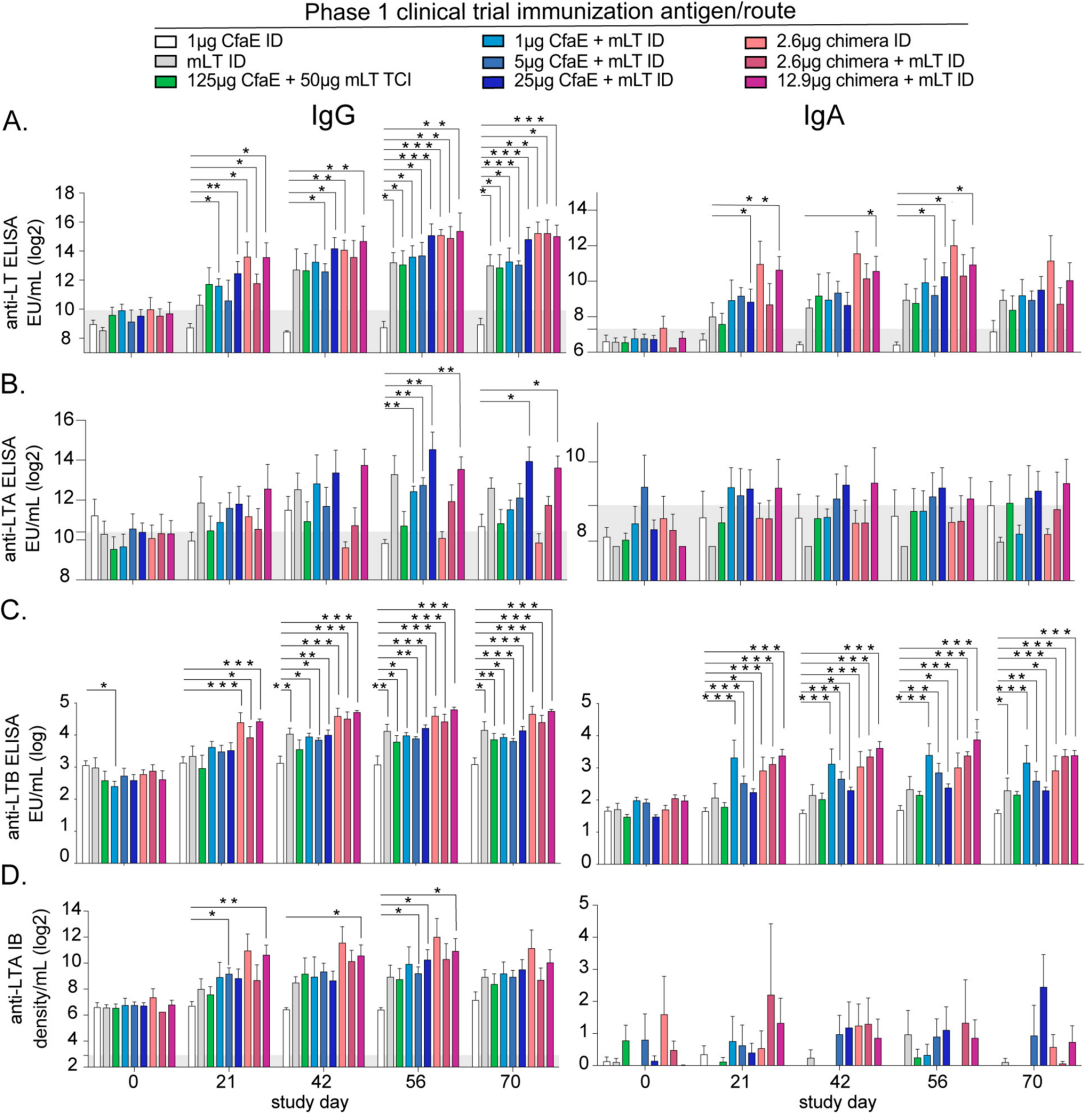
LT toxoid antigens drive magnitude of anti-LT or subunit serum and IgG responses post-vaccination. Phase 1 clinical trial serum analyses of LT or subunit Abs organized by IgG (left) or IgA (right) by study day with immunization groups indicated from **A** Bar graph of serum anti-LT Abs ELISA (reported as log2 ELISA units per ml (EU/ml)) by study day. **B** Bar graph of serum anti-LTA Abs ELISA (log2 EU/ml) by study day. **C** Bar graph of serum anti-LTB Abs ELISA (log10 EU/ml) by study day from historical ELISA data (from [39]). Note that the previous anti-LTB IgA assay has high levels of IgG cross-reactivity than the other LT or LTA IgA assays. **D** Bar graph of serum anti-LTA Abs Immunoblot (IB log2 band density per ml) by study day. Bars at mean + SEM with significance indicated using two-way ANOVA with uncorrected Fisher’s LSD post-test to the 1 μg CfaE ID group (lacking any LT toxoid antigen) indicated as *P < 0.05, **P < 0.01, or ***P < 0.001. Gray area indicates the limit of detection for each assay

**LT Abs are dependent upon antigen dose, delivery route, and exhibit binding to both subunits in Phase 1 clinical trial samples.** We first profiled the responses to LT toxin in human serum days 0–70 during a Phase 1 Clinical Trial ([41], Fig. 1A). In this study, groups of volunteers (n = 4–9) were vaccinated using combinations of a prototype ETEC adhesin antigen, dscCfaE (CfaE), or an adhesin-toxoid antigen, dscCfaE-CTA2/LTB5 (chimera), with or without mLT by TCI or ID vaccination. Serum samples were tested for anti-toxin Abs or functional neutralizing Ab by directly measuring in vitro accumulation of cAMP (Fig. 1C, 2, Fig.S2).

These analyses revealed significant heterogeneity in antibody responses to LT toxin subunits, driven in part by the type of toxoid vaccine antigen (chimera or only mLT) and the dosage of adhesin antigen. Specifically, all vaccination groups except mLT ID developed neutralizing antibodies after 3 immunizations on day 56 or 70 compared to subjects receiving CfaE ID. The 12.9 μg chimera + mLT ID group, which corresponds to approximately 8 μg of LTB, was the only group that developed neutralizing antibodies after one immunization on day 21 (Fig. 1C). After two vaccinations on day 42, all chimera groups had significantly increased LT neutralization. Detection of anti-LT or -LTB IgG by ELISA largely followed these trends (Fig. 2A,C). Note that little anti-LTB IgG was detected by IB method and thus not quantified (Fig.S2). All groups developed high levels of persistent anti-LT or -LTB IgG antibodies (including mLT ID) based on day 56 and day 70 detection post-third immunization. More rapid increase of anti-LT IgG was detected in CfaE + mLT ID immunization groups (e.g. on day 21) than with the neutralization assay (Fig. 2A). This likely reflects the differences in functional neutralizing antibodies versus antigen-binding antibodies.

Abs to LTA were detected using both ELISA and IB, within distinct timeframes: linear epitope Abs (e.g. IB method) were evident on days 21–56, while conformational Abs (e.g. ELISA) were detected on days 56–70 (Fig. 2B,D). This suggests both maturation of the B-cell germinal center responses and unique presentation of the A-subunit which undergoes unfolding intracellularly to move through the sec61 pathway in the endoplasmic reticulum to the cytosol. As expected, mLT-containing formulations were critical to the development of these anti-LTA Abs because the other formulations contain no A-subunit antigens. Unexpectedly the higher dose of companion antigen (chimera, CfaE) enhanced the levels of anti-LTA IgG. Thus, the higher doses of a companion immunogen along with mLT facilitates development of a robust, long-lasting antibody response to the entirety of the holotoxin.

TCI vaccination failed to elicit consistent levels of anti-LTA IgG and IgA Abs and led to only modest levels of functional neutralization Abs by day 70 (Figs. 1C,2). This was true even with co-administration of 50 μg mLT, 500 times higher than the 0.1 μg dose used for ID delivery. Interestingly, the same dose was well-tolerated and immunogenic in preclinical models with mice [46] and guinea pigs. This observation strongly suggest that, while TCI was successful in delivering large doses of the holotoxin as an ETEC vaccine candidate in the past[47], it is less efficient to deliver a vaccine formulation with a subunit adhesin adjuvanted by mLT at the doses and methods used here and elsewhere[48].

Serum IgA is often used as an indicator of mucosal immunity and thought to be a critical component of gastrointestinal host defense. Robust and rapid detection of IgA antibodies like that observed with anti-LTB IgA (the previously reported responses that utilized a more IgG cross-reactive secondary antibody) was not observed even with anti-LT IgA ELISA (Fig. 2, right side). However, between day 21 and 56, immunizations containing higher doses of CfaE or chimera + mLT ID had significant anti-LT IgA expression compared to CfaE ID. The lack of anti-LT IgA detection at study day 70 may indicate transient IgA plasma cell secretion or homing of IgA-secreting cells from the periphery into mucosal tissues.

**Fig. 3:**
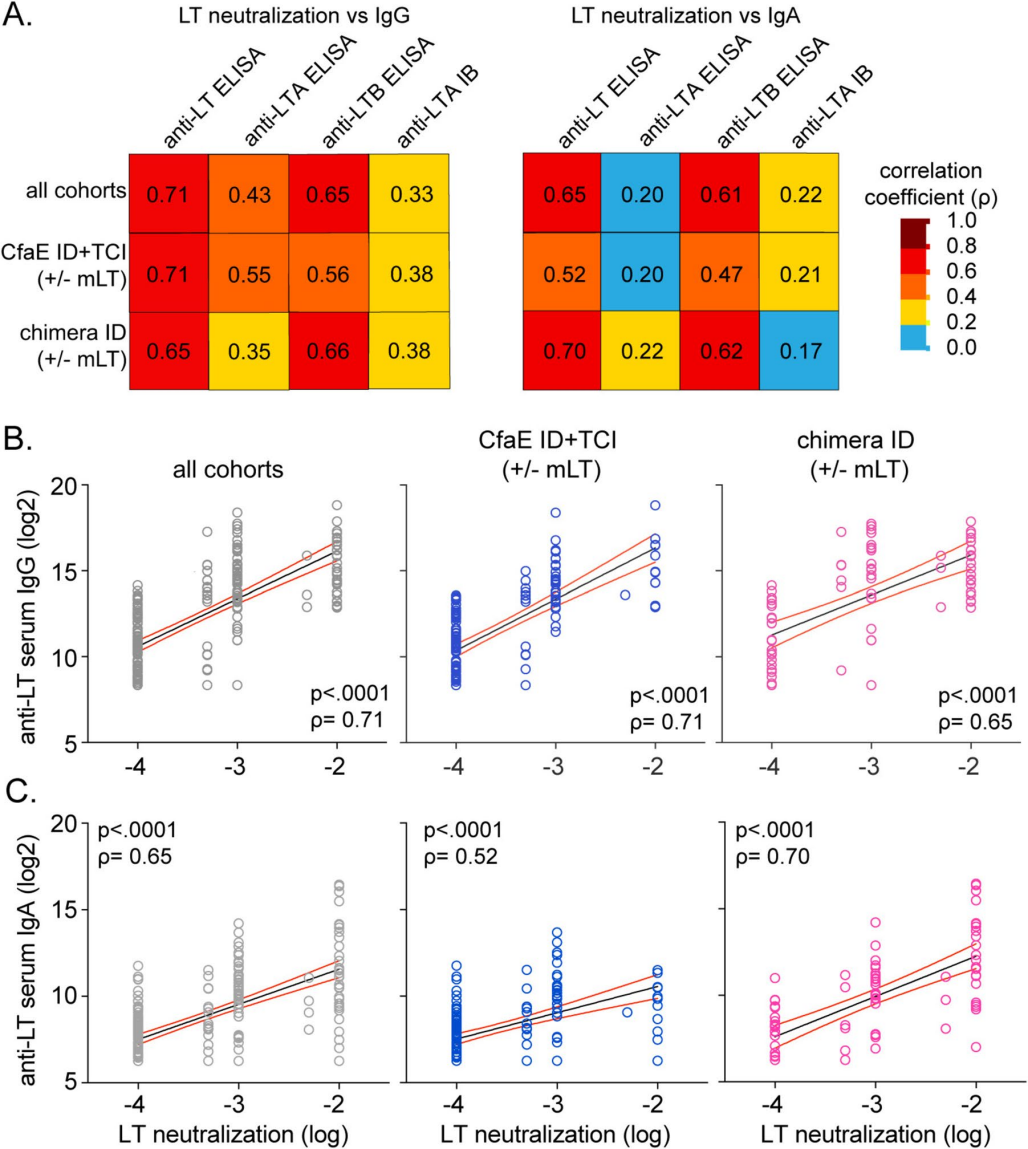
Phase 1 serum anti-LT IgG and IgA correlate best to LT neutralization. **A** Correlation heat maps between serum LT neutralization vs. anti-LT Abs IgG (left) and IgA (right) assay estimated by the REML method and are reported as Spearman’s rank correlation coefficient as indicated. All correlations are significant at P < 0.001. Color and number indicate correlation coefficient. Rows are organized by LT toxoid antigen including all cohorts, CfaE ± mLT ID or TCI, and chimera ± mLT ID. **B** Graphs of select IgG correlations with individual subjects depicted as symbols. **C** Graphs of select IgG correlations with individual subjects depicted as symbols

**LT Abs correlate well to toxin neutralization in Phase 1 clinical trial samples.** To determine if one method of LT Abs detection was superior to another in predicting Abs functionality, we correlated IgG and IgA Abs to toxin neutralization (Fig. 3, Table S3). All assays were significantly (P < 0.001) correlated to toxin neutralization for all vaccine groups, CfaE antigen vaccine groups, or chimera antigen vaccine groups. However, anti-LT IgG had the highest correlation to LT neutralization (ρ = 0.65–0.71, Fig. 3A-B). Interestingly, anti-LT IgA was also correlated with neutralization (ρ = 0.52–0.70, Fig. 3A, C). The CfaE groups’ Abs exhibited equivalent correlation coefficient between anti-LTA IgG (ELISA) and anti-LTB IgG to LT neutralization (respectively, ρ = 0.55 and 0.56). While the chimera groups exhibited a stronger correlation of anti-LTB IgG than anti-LTA IgG (respectively ρ = 0.66 and 0.35), consistent with the high LTB antigen dose within the chimera. Thus, we conclude there is evidence that antibodies against toxin or its subunits promote toxin neutralization but that the relative ratio of these Abs is driven by the nature of the toxoid antigens selected for immunization.

**Fig. 4:**
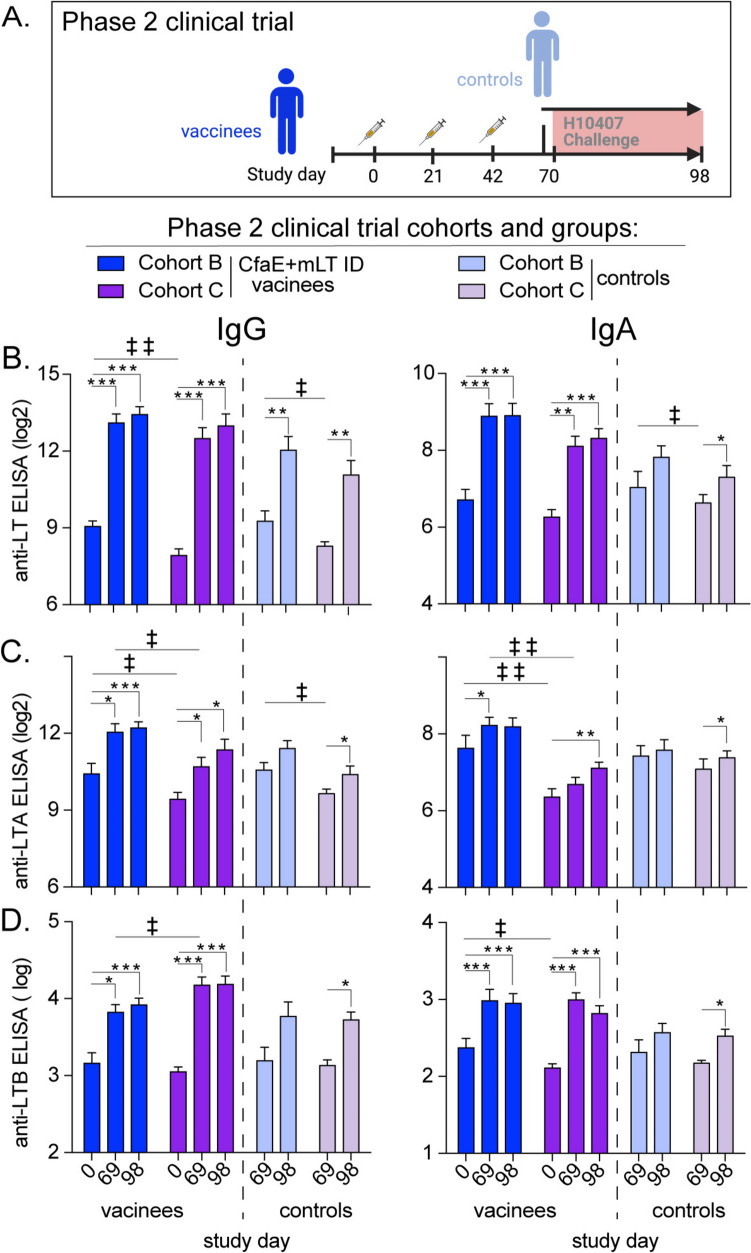
Phase 2 LT serum antibody analyses between vaccinees or controls pre- and post-H10407 challenge. **A** Schematic of Phase 2 clinical trial cohorts (A, B and C) that were immunized with 25 ug CfaE + 0.1 ug mLT on days 0, 21, and 42. They were then challenged with oral H10407 on day 70. Cohort A did not reach correct inoculum for challenge so not selected for these additional Abs analyses. Unvaccinated controls for each cohorts were recruited on day 69 and then challenged. Groups/cohort contained 9–16 subjects. (B) Bar graph of serum anti-LT IgG (right) or IgA (left) ELISA (log2 EU/ml) by study day. (**B**) Bar graph of serum anti-LTA Abs tested by ELISA (log2 EU/ml) by study day. (**C**) Bar graph of serum anti-LTB Abs ELISA (log10 EU/ml) by study day from historical ELISA data (from [39]). Note that the previous anti-LTB IgA assay has high levels of IgG cross-reactivity than the other LT or LTA IgA assays. Bars at mean + SEM with significance indicated for vaccinees using paired one-way Anova with Dunnett post-test indicated as *P < 0.05, **P < 0.01, or ***P < 0.001 or for controls using paired T-test two-way indicated as ‡ < 0.05 and ‡ ‡ < 0.01

**Comparison of the serum anti-LT Abs responses after CfaE + mLT ID vaccine to oral ETEC challenge outcomes using Phase 2 clinical trial samples.** The overall results and safety profile of the Phase 1 clinical trial discussed above led to the 25 μg of CfaE delivered with 0.1 μg of mLT ID being further investigated for preliminary efficacy in a controlled human infection model (CHIM) in three cohorts (A,B,C). Following vaccination on days 0, 21, and 42 vaccine participants along with unvaccinated controls were orally challenged with CFA/I^+^ LT^+^ ST^+^ ETEC strain H10407 on day 70 ([43], Table S2). The parent study evaluated data in aggregate across cohorts for adverse events, CfaE and LTB immunogenicity and challenge outcomes and concluded that despite differences in attack rates between cohorts, this was the first study to demonstrate protection against ETEC challenge after intradermal vaccination. Here we looked closer at the cohort immune responses due to an aberrant naïve attack rate in cohort A, ony samples from participants in cohorts B and C, where the naïve attack rates were consistent (e.g., MSD > 50%) were analyzed here (n = 9–17/group, Fig. 4A).

We first quantified IgG and IgA Abs to LTA and LT holotoxin at day 0, day 69 (post-vaccination and/or pre-challenge), or day 98 (four weeks post-challenge) in addition to previously reported anti-LTB IgG and IgA[43]. Our analyses revealed development of antibody responses to the toxin and its subunits (Fig. 4B-D) with individual subject variability detected (Fig.S3). Vaccination resulted in a significant increase in detection of anti-LT, LTA, and LTB IgG and anti-LT IgA Abs by day 69 irrespective of cohort. We observed a difference between vaccinees in detection of serum anti-LTA IgA in cohort B at day 69 and not in cohort C (Fig. 4C). Vaccinees developed significant anti-LTA IgA post-challenge on day 69 or day 98 compared to day 0 with cohort specific differences. Challenge in unvaccinated controls resulted in significant increase in anti-LT IgG (all cohorts). However, only cohort C unvaccinated controls developed significantly higher antibody responses at day 98 post-challenge to other subunits and IgA compared with pre-challenge serum, likely reflecting altered conditions of H10407 experimental challenge or host factors (pre-existing immunity or demographics) between cohorts.

**Fig. 5:**
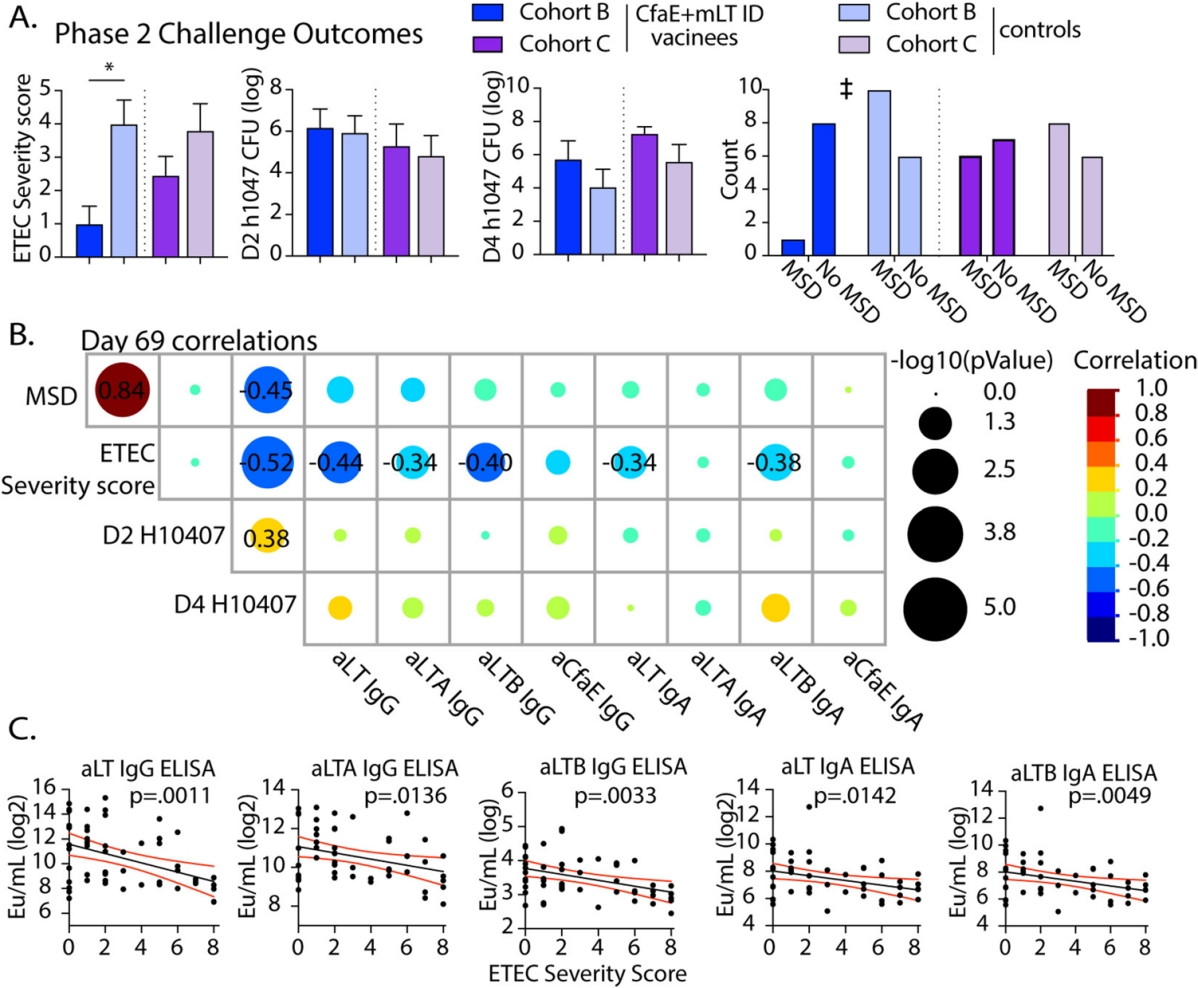
Analyses of Phase 2 H10407 Challenge outcomes with Day 69 antibodies. (**A**) Bar graphs of challenge outcomes with comparisons made between vaccinated and control groups for cohorts B and C. Comparisons were done by one-way ANOVA with Kruskal–Wallis post-test indicated as *P < 0.05 for ETEC Severity Score and H10407 CFUs. A 2 × 2 contingency table was used to compare MSD outcomes between vaccinated and control groups indicated as ‡P < 0.05. (B) Heat map depicting correlations (Spearman’s Rank) and significance of challenge outcomes and Day 69 (pre-challenge) antibody levels for cohorts B and C. Colors represent the correlation and the size of circles indicate significance. Correlations displayed for significant comparisons. **C** Linear regressions of selected significant antibody and ETEC severity score relationships with subjects depicted as symbols

Next, we compared the anti-toxin Ab responses developed post-vaccination and their correlation to outcomes post-challenge*.* While MSD has been a traditional primary outcome, here we focused correlation analyses on the relationship to ETEC severity score, which is a continuous measure developed to assess the spectrum of diarrheal outcomes and clinical signs of disease in response to challenge[49]. Individual cohorts had distinctive outcomes post-challenge (e.g. ETEC severity score, MSD, fecal day 2 [D2] or day 4 [D4] H10407 bacterial CFUs, Fig. 5A). These D2 and D4 fecal bacterial CFUs corresponded to study day 72 and 74, respectively. These outcomes correlated significantly to each other except for D2 H10407 CFUs (Fig. 5B**,**), which likely reflects excretion of initial challenge dose. Neither subject sex, race, or age were significantly related to challenge outcomes, though this study was not powered to make these comparisons.(Table S4, S5). We performed correlation analysis of toxin antibody responses in both vaccinees and controls on day 69 prior to ETEC challenge. Importantly, toxin IgG (anti-LT, -LTA, -LTB) and LT IgA significantly and inversely correlated to ETEC severity score, with the highest correlation coefficient observed for LT IgG (ρ = − 0.52, Fig. 5B). No significant correlations were observed with adhesin-specific immunity or anti-CfaE IgG or IgA. Similar responses were observed with linear regressions (Fig. 5C) and Abs comparison to MSD outcomes (Fig.S3).

**Fig. 6:**
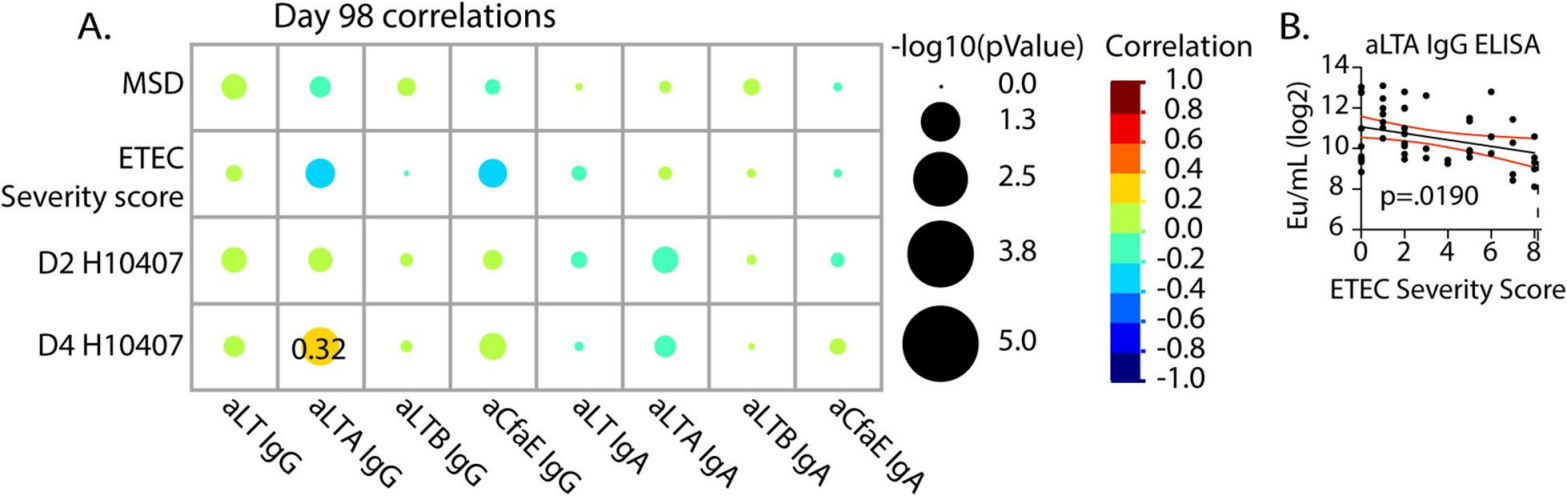
Correlation analyses of Day 98 antibodies data with post challenge outcomes. **A** Heat map depicting correlations (Spearman’s Rank) and significance of challenge outcomes and Day 98 (4 weeks post-challenge) antibody levels for cohorts B and C. Colors represent the correlation and the size of circles indicate significance. **B** Linear regressions of anti-LTA IgG and ETEC severity score with subjects depicted as symbols

We also performed similar analyses with day 98 post-challenge Abs responses (Fig. 6, Fig.S4). The only significant relationship observed was higher levels of anti-LTA IgA post-challenge at day 98 that correlated with higher D4 H10407 fecal bacterial burden and by linear regression to ETEC severity scores (Fig. 6A, B, Table S5). This suggests that individuals able to mount a rapid anti-LTA IgG response during challenge may help protect them from severe disease.

## Discussion

The objective of this study was to perform a more detailed examination of the antibody responses to LT toxin and subunits to better understand recent clinical trials and inform selection of ETEC vaccine candidates. Using the Phase 1 and Phase 2 clinical trial samples from recent ETEC vaccination with adhesin and toxoid subunit vaccination by TCI or ID delivery, we were able to evaluate and compare toxin antibody assays between ETEC vaccine candidates and CHIM cohorts. Among all the methodologies we employed, anti-LT IgG ELISA correlated most strongly to the toxin neutralization assay or ETEC challenge outcomes. This indicates this simple ELISA technique is an excellent assay to quantify the toxin responses and predict protection against LT + or LT + ST + ETEC strains. Importantly, though regional strain differences in toxin expression are evident in cross country comparisons, these data confirm older studies [4, 11–14] to once again define the importance of vaccine-driven anti-LT antibodies, even when generated by parenteral injection, as a major mechanism of protection against diarrheal disease from the two-thirds of ETEC strains that express LT or both LT and ST[4, 7–10].

In our Phase 1 analyses, groups receiving LT developed toxin neutralizing serum Abs over the course of the study, observable as soon as after the first vaccination and frequently peaked after the completion of the vaccination series. The fastest development of serum neutralizing Abs occurred following vaccination with chimera, likely due to the large LTB content, and following vaccination with 25 μg of CfaE + mLT, which indicates an important interplay between the co-administered protein dose and the toxoid adjuvant. This was apparent by serum samples exhibiting toxin neutralization ability appearing by day 21 after the first vaccination.

Our analyses revealed the development of antibody responses to LT and its subunits with individual variability by subject and cohort. In the Phase 2 study, vaccination with CfaE + mLT resulted in a significant increase in the detection of anti-LT, LTA, and LTB IgG and IgA Abs though subject variability was observed in our supplemental analyses. Importantly, pre-existing toxin immunity and memory responses likely contributed to cohort difference in ETEC H10407 challenge outcomes. Among unvaccinated control subjects, those in cohort C developed significant antibody responses post-challenge including anti-LT, LTA, and LTB IgG and IgA Abs. However, the control subjects in cohort B only developed significant serum anti-LT IgG Abs. Comparisons of baseline sera levels shows that the cohort B controls had significantly higher pre-existing toxin immunity (aka day 69 anti-LT IgG, IgA and ani-LTA IgG) than controls in cohort C.

No vaccine or control group was protected from acute H10407 infection by day 2 or 4 post-challenge based on detection of bacteria in fecal specimens. However, protection from clinical outcomes (MSD or ETEC severity) was observed with vaccination particularly in cohort B. We also confirmed the advantage of using the ETEC severity scoring range than the categorical MSD variables in relationship to immunogenicity pre-challenge. To more clearly compare Abs measures against the AB_5_ structure of LT or its subunits (irrespective of pre-existing or vaccine-derived), we correlated individual serum Abs to clinical or infection outcomes following oral challenge with the CFA/I + LT + ST + ETEC strain H10407. For day 69 (pre-challenge), anti-LT IgG ELISA data was the best indicator of protection, even over anti-CfaE antibodies. As H10407 expresses both secretory toxins, this provides evidence that vaccine-induced antibodies to LT can provide some protection against ST + LT + ETEC strains even without an ST toxoid antigen. However, four weeks after the challenge, the correlations of antibody responses were altered. Specifically, positive linear relationships no longer existed between anti-LT IgG and the other Abs tested (although these were still significantly correlated) and anti-LTA IgA was associated with protection. This is interesting considering the generation of anti-LTA Abs after human infection or animal vaccination have protective abilities [15, 16]. In addition, ETEC isolates also exhibit variability in LT-type toxins (e.g. Type I, Type IIa, Type IIb), particularly in animal infections, and these toxin types are reportedly more similar in their A-subunit than B-subunit by amino acid analyses[38–40]. It may be that broad protection against ETEC LT-type toxins may require anti-LTA Abs and this could be particularly important for pig or other veterinary ETEC vaccines. It is also tempting to speculate that this may have played a role in the failure of a LT only skin vaccine in Phase 3 clinical trials [33], as skin vaccination in the Phase 1 study of our analyses did not generate strong anti-LTA Abs.

Limitation and future directions of this study suggested by our analyses include a more detailed serological analysis that includes other isotypes (IgG1-4, IgA1, IgA2, etc.) and antibody functionality beyond toxin neutralization. In addition, a more detailed analyses of which Abs-binding locations are optimal within the amino acid locations of the A- and B-subunits show best ability for stable binding and inhibition of toxin activities, which could also support development of therapeutics including monoclonal antibodies. Lastly, our study lacked any evaluation of mucosal specific immunity or antigen-specific T-cell responses in these cohorts which likely are contributors to protective immunity.

We therefore conclude that strategies generating and measuring immunity to the complete AB_5_ structure of LT and subunits are better determinant of assessing protective immunity against acute LT + or LT + ST + ETEC diarrheal secretion in humans. Furthermore, pre-existing Abs to LT toxins AB_5_ structure and/or toxin A-subunit may play unappreciated roles in ETEC protection in vaccine-naïve populations.

## Supplementary Information

Below is the link to the electronic supplementary material.

Supplementary file1 (XLSX 21 KB)

Supplementary file2 (DOCX 965 KB)

## Data Availability

Data reported in the manuscript is available from the corresponding author upon reasonable request.
