# Supplementary material for: Protective antibodies against enterotoxigenic Escherichia coli are generated from heat-labile toxoid vaccination and exhibit subject- and vaccine-specific diversity
Source: Med Microbiol Immunol. 2025 Feb 11;214(1):10. doi: 10.1007/s00430-025-00817-3 (PMC11814043; doi:10.1007/s00430-025-00817-3)
Supplement: Supplementary file 2 — Supplementary file2 (DOCX 965 KB) [file 430_2025_817_MOESM2_ESM.docx]

Supplemental Material for: Protective antibodies against enterotoxigenic *Escherichia coli* are generated from heat-labile toxoid vaccination and exhibit subject- and vaccine-specific diversity.

**Table S1: ETEC Phase 1 Clinical Trial Immunization Groups & Tested Samples (ClinicalTrials.gov Identifier: NCT01644565).**

| **Study Group Details – ETEC Clinical Trial**  *(WRAIR Spring/Summer 2013)* | | | | **Serum Samples Used for Additional Analysis** | | | | | |
| --- | --- | --- | --- | --- | --- | --- | --- | --- | --- |
| **Cohort** | **Group** | **Route/Immunization antigens** | **#**  **per Group** | **Day**  **0** | **Day 21** | **Day 42** | **Day 56** | **Day 70** | **All** |
| A | A-1 | Intradermal 1μg dscCfaE | 5 | 5 | 5 | 5 | 5 | 5 |  |
|  | A-2 | Intradermal 2.6μg chimera | 5 | 5 | 5 | 5 | 5 | 5 |  |
|  | A-3 | Intradermal 0.1μg mLT | 5 | 4 | 4 | 4 | 4 | 4 |  |
| B | B-1 | Intradermal 1μg dscCfaE  + 0.1μg mLT | 4 | 4 | 4 | 3 | 3 | 4 |  |
|  | B-2 | Intradermal 2.6 μg chimera  + 0.1 μg mLT | 5 | 5 | 4 | 4 | 4 | 4 |  |
| C | C-1 | Intradermal 5μg dscCfaE  + 0.1μg mLT | 7 | 4 | 4 | 4 | 4 | 4 |  |
|  | C-2 | Intradermal 12.9μg chimera + 0.1μg mLT | 7 | 7 | 7 | 7 | 7 | 7 |  |
| D | D-1 | Intradermal 25μg dscCfaE  + 0.1μg mLT | 9 | 9 | 9 | 7 | 7 | 8 |  |
|  | D-2 | Transcutaneous 1.25 mg dscCfaE + 50 μg mLT | 7 | 6 | 6 | 5 | 5 | 5 |  |
| Totals | | | | 49 | 48 | 44 | 44 | 46 | **231** |

**Figure S1. Anti-Human IgA reactivity to recombinant IgG or IgA standards by ELISA.** Results of ELISA testing to confirm cross-reactivity of human detection antibodies to human recombinant IgG or IgA proteins. Cross-reactivity of antibodies were determined using standard ELISA protocols with coating dilutions as shown on x-axis of graphs with recombinant human IgG (Pierce 31154**,** Sigma 12511) or human IgA (SouthernBiotech, lambda 0155L or Kappa 0115K) noted as standard (std). This was followed by washing and detection using anti-human IgA-biotin from SouthernBiotech 2050-08 (top graph) or KPL 16-10-01 bottom graph) and then anti-biotin-AKP (Sigma A7064). Cross reactivity was calculated <0.3% for anti-IgA from SouthernBiotech vs 50% using anti-IgA frοm KPL against human IgG we determined by averaging OD values for the standard concentrations (between 1000ng – 0.01) by % of OD detected using IgA standards.

A.


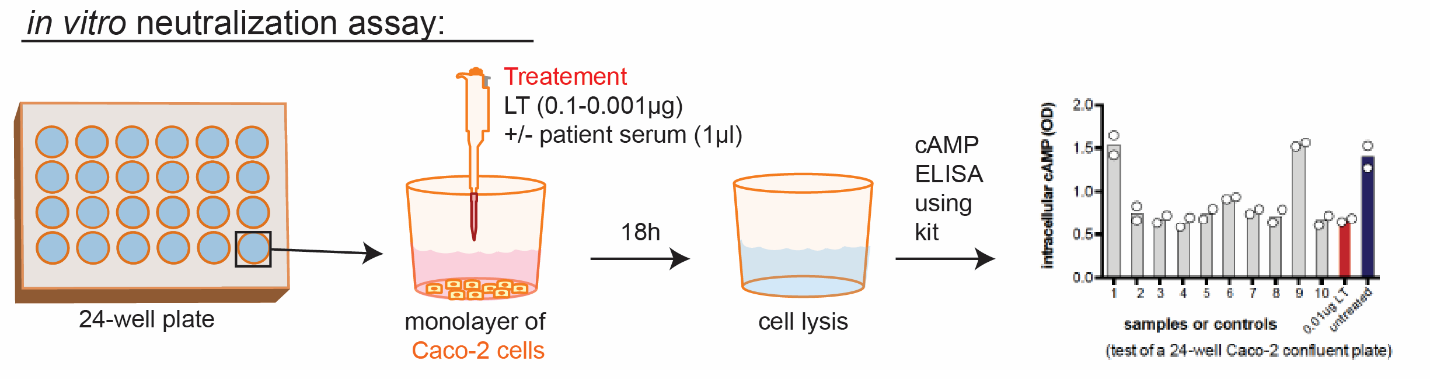


B.


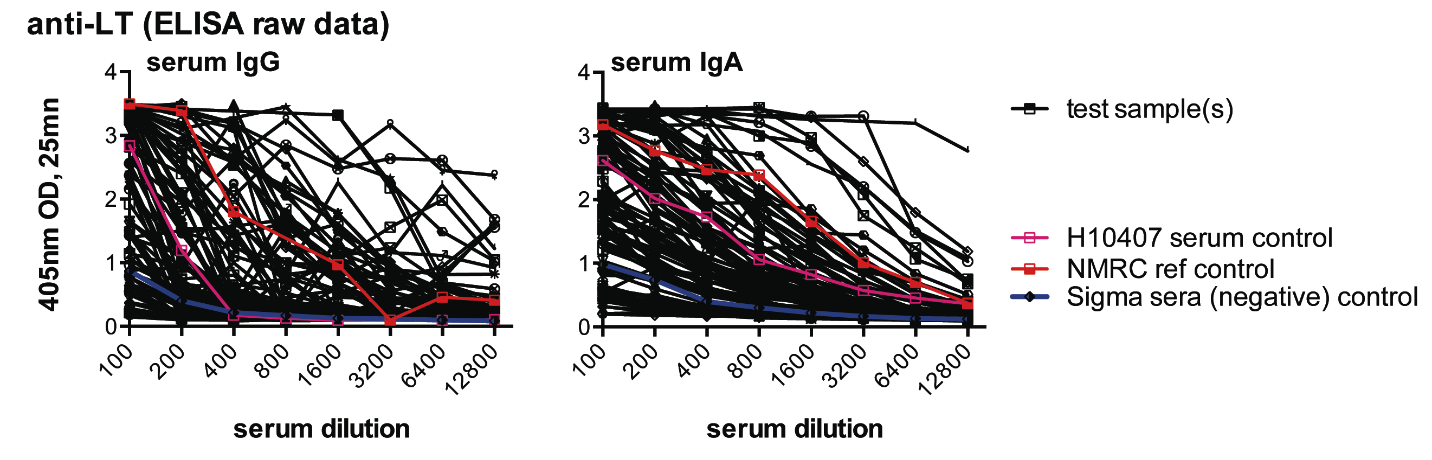


C.


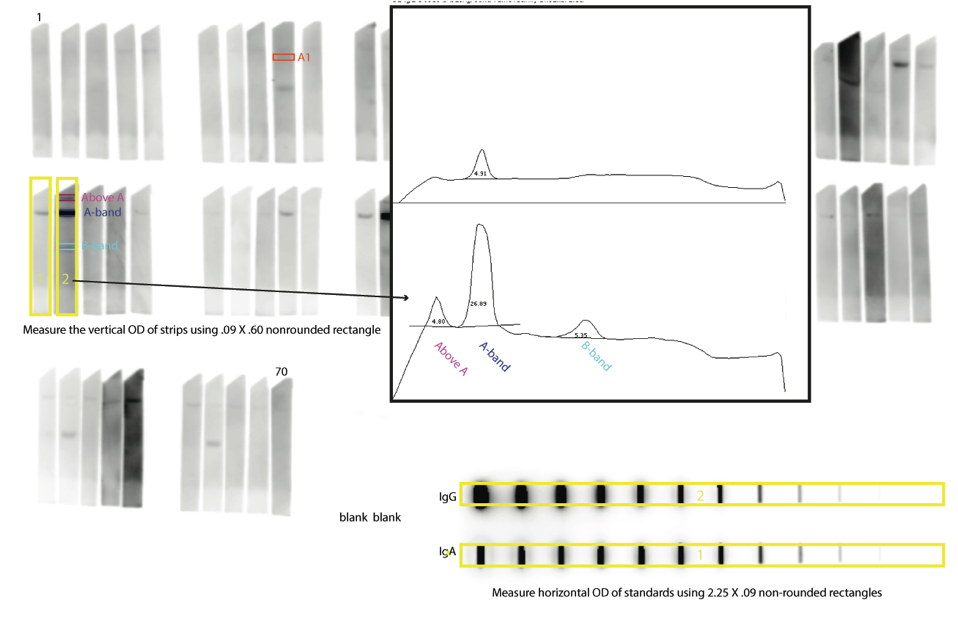


**Supplemental Figure 2. Example data from individual assays used to test ETEC Phase 1 clinical trial samples.** A. Diagram of the *in vitro* LT neutralization assay process and example raw data. B. Raw data from anti-LT IgG and IgA ELISA including individual test samples (black) by dilution factor. Control serum was included in each ELISA plane including H10407 serum control, NMRC ref control, and Sigma sera (colored lines). C. Example of LT immunoblots with A- and B-band analyses. Each strip was proved with test or control sample and developed as a group with a set of human IgG and IgA standards as shown. Blots were analyzed for A- and B-band Ig density/ mL serum with background subtraction as shown.

**Table S2.** Tested serum samples from Phase 2b ETEC Clinical Trial Cohorts*

| **Phase 2 ETEC Clinical Trial Cohorts** | | | **Serum Collection Time Points** | | |
| --- | --- | --- | --- | --- | --- |
| Cohort | Group | Number per Group | Day 0 | Day 69 | Day 98 |
|  |  |  | (pre-vaccination) | (post-vaccination, pre-challenge) | (post-challenge) |
| A** | Vaccinated | 19 | 19 | 19 | 19 |
|  | Naïve controls | 11 | *n/a* | 11 | 11 |
| B | Vaccinated | 9 | 9 | 9 | 9 |
|  | Naïve controls | 17 | *n/a* | 17 | 17 |
| C | Vaccinated | 13 | 13 | 13 | 13 |
|  | Naïve controls | 15 | *n/a* | 15 | 15 |
|  | | Total Samples: | 79 (cohort 1) + 61 (cohort 2) + 69 (cohort 3) | | |
|  | | | | | |

* Vaccination consists of 25 ug dscCfaE + 0.1 μg LT(R192G) delivered intradermally on days 0,21,42. CFA/1+ ETEC (H10407) oral challenge was conducted on day 70

** Cohort A was not included in tested samples


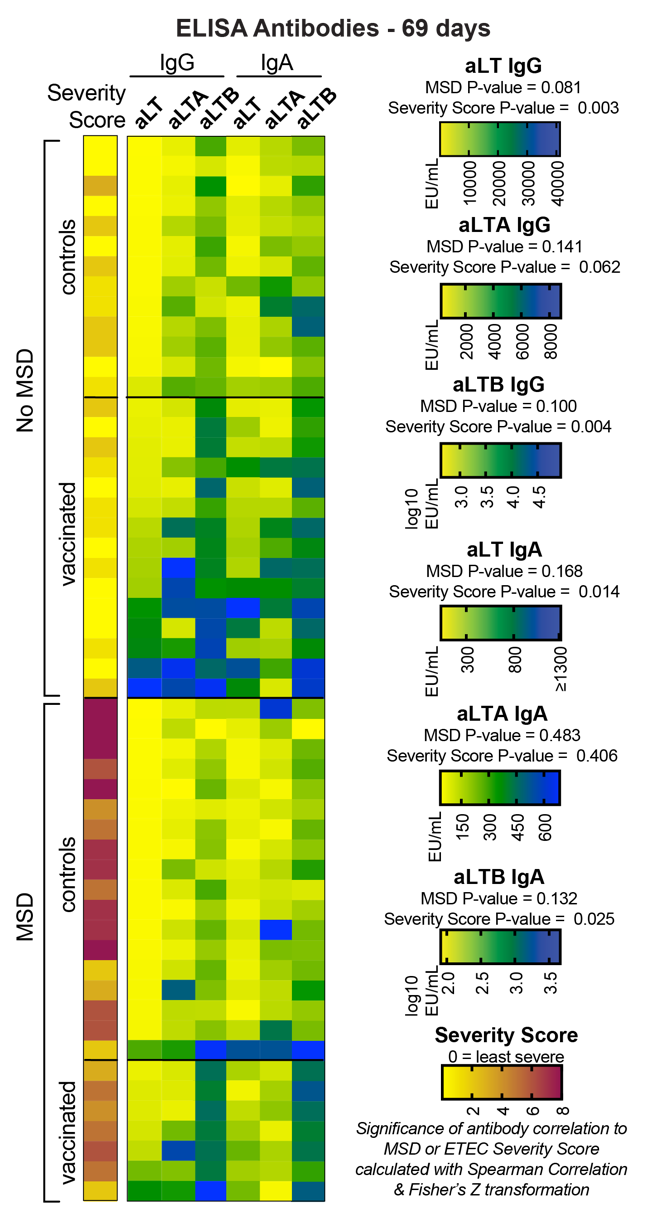


**Figure S3. Heat map of assay results organized by moderate-to-severe (MSD) diarrheal outcome post-challenge for (pre-challenge/post-vaccination) day 69 serum analyses.** ELISA results from day 69 sample testing were organized in a heat map grouped by controls and vaccinated subjects within those exhibiting MSD or No MSD symptoms post-H10407 challenge (previously collected NMRC data) with each subject’s ELISA antibody results and ETEC Severity Score organized across the row. *P-*values indicate the significance of Spearman Correlation with Fisher’s Z-transformation for each ELISA antibody compared to MSD or Severity Score, as indicated. The probability of not developing MSD post-H10407 challenge in all tested subjects was related to antibody responses pre-challenge (day 69), including anti-LT IgG (P=0.128), anti-LTA IgG (P=0.142), anti-LTB IgA (P=0.146), and anti-LTB IgG (P=0.191). Anti-LT IgA or anti-LTA IgA Abs were not associated with not developing MSD following H10407 challenge.


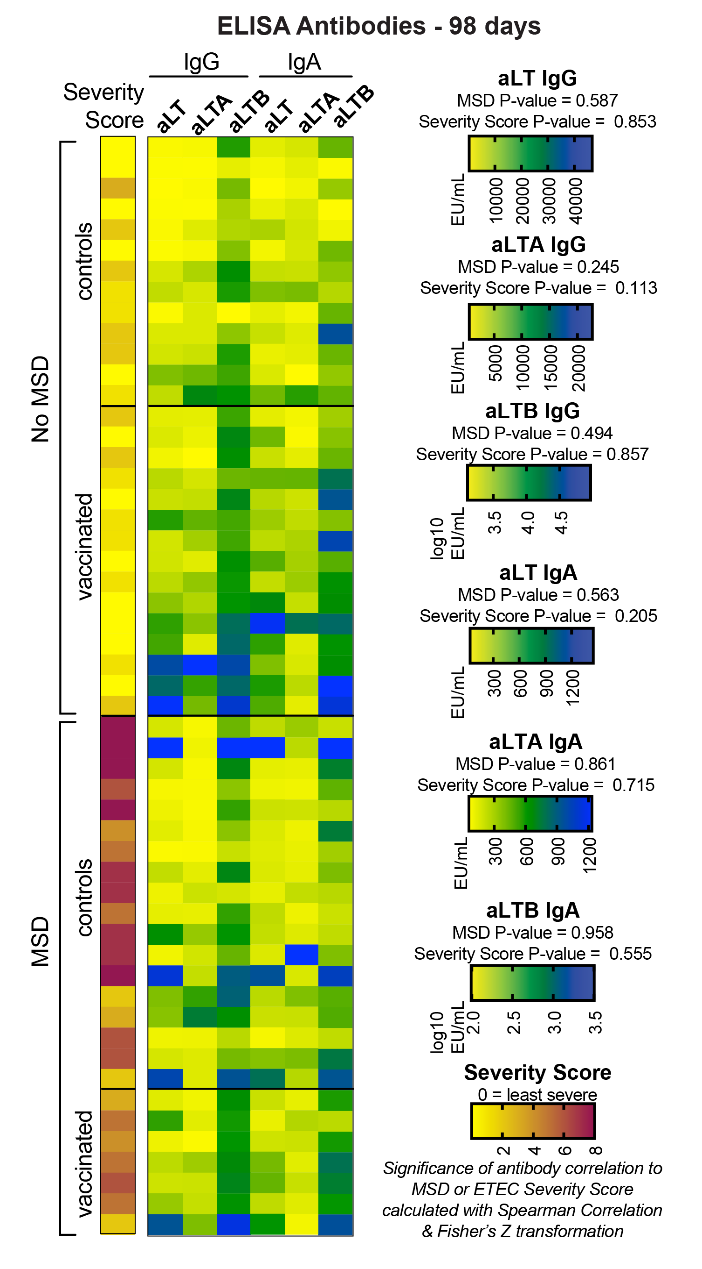


**Figure S4. Heat map of assay results organized by moderate-to-severe diarrheal (MSD) outcome post-challenge for (post-challenge) day 98 serum analyses.** ELISA results from day 98 sample testing were organized in a heat map grouped by controls and vaccinated subjects within those exhibiting MSD or No MSD symptoms post-H10407 challenge (previously collected NMRC data) with each subject’s ELISA antibody results and ETEC Severity Score organized across the row. *P-*values indicate the significance of Spearman Correlation with Fisher’s Z-transformation for each ELISA antibody compared to MSD or Severity Score, as indicated.
